# Predicting Seasonal Performance in Professional Sport: A 30-Year Analysis of Sports Illustrated Predictions

**DOI:** 10.3390/sports9120163

**Published:** 2021-12-01

**Authors:** Justine Jones, Kathryn Johnston, Lou Farah, Joseph Baker

**Affiliations:** Department of Kinesiology and Health Science, York University, Toronto, ON M3J 1P3, Canada; justinejones14@gmail.com (J.J.); loufar@yorku.ca (L.F.); bakerj@yorku.ca (J.B.)

**Keywords:** prediction, futures betting, professional sport, sport forecasting

## Abstract

In 2017, Sports Illustrated (SI) made headlines when their remarkable prediction from 2014 that the Houston Astros (a team in one of the lowest Major League Baseball divisional rankings) would win the World Series, came true. The less-publicised story was that in 2017, SI predicted the Los Angeles Dodgers to win the Major League Baseball (MLB) title. Assessing the forecasting accuracy of experts is critical as it explores the difficulty and limitations of forecasts and can help illuminate how predictions may shape sociocultural notions of sport in society. To thoroughly investigate SI’s forecasting record, predictions were collected from the four major North American sporting leagues (the National Football League, National Basketball Association, Major League Baseball, and National Hockey League) over the last 30 years (1988–2018). Kruskal–Wallis H Tests and Mann–Whitney U Tests were used to evaluate the absolute and relative accuracy of predictions. Results indicated that SI had the greatest predictive accuracy in the National Basketball Association and was significantly more likely to predict divisional winners compared to conference and league champions. Future work in this area may seek to examine multiple media outlets to gain a more comprehensive perspective on forecasting accuracy in sport.

## 1. Introduction

In 2014, Sports Illustrated (SI) made the lofty prediction that the Houston Astros (a team in one of the lowest MLB divisional rankings) would win the 2017 World Series. Three years later, to the public’s surprise, this prediction came true. The media marvelled at this occurrence, giving credit to SI’s predictive accuracy [1,2]. The less-publicised story, however, was that the team SI predicted in 2017 to win the 2017 Championship was the Los Angeles Dodgers. Surprisingly, SI was praised for the accuracy of their 2014 prediction, not criticised for changing that prediction to one that was incorrect in the same year. The original prediction was especially odd since the Astros were at the bottom of the league standings when the prediction was made. It is possible that the bizarreness of this prediction (moving from last to first place) may have influenced how it was perceived and evaluated [3,4,5,6]. However, these differing forecasts highlight the complexities of talent identification and prediction decisions in sport. In this study, we examine the accuracy of SI’s pre-season predictions across the four major North American professional sports leagues—Major League Baseball (MLB), the National Football League (NFL), the National Basketball Association (NBA), and the National Hockey League (NHL) over the last 30 years.

Since the turn of the century, the sports industry has seen a rise in the use of analytics [7]. A large catalyst for this shift was the Major League Baseball (MLB) team Oakland Athletics’ ‘Moneyball’ movement where the team experienced tremendous success (second place at the World Series) despite financial restrictions, by using objective measures and statistics [8]. This phenomenon raised awareness of the value of using quantitative approaches to minimise biases from more subjective approaches to decision-making. Currently, computer technology and statistical acumen occupy a growing role in the development of accurate forecasting models and although expert opinions have been evaluated in fields like meteorology [9] and finance [10] there has been less attention to the accuracy of experts in predicting sport outcomes [11,12]. There is, however, an exception with recent years bringing new light to evaluating the expert and non-expert (also referred to as ‘crowd’) forecasts in sports. Specifically, new research published includes topics such as (i) evaluating expert forecasters over an extended period of time [13,14], (ii) evaluating crowds of tipsters (or semi-expert forecasters) [15,16], (iii) evaluating the informational content of sports experts through their contributions on social media [17], and (iv) evaluating semi-expert judgments regarding sporting predictions to improve forecast accuracy [18]. Assessing the forecasting accuracy of ‘experts’ is critical as it explores the difficulty and limitations of forecasts and challenges us to re-think notions of athlete development and the path to elite performance.

Evidence comparing the accuracy of expert opinions and computer-generated models is varied. On the one hand, there is a substantial body of literature showing the benefits of removing the ‘human-element’ of the decision-making process [8,19,20] while on the other hand, there is evidence highlighting the importance of the human ‘eye’ in judgements [21,22]. For example, it was discovered that on average, computer-based models made more correct forecasts at the 2003 Rugby Union World Cup than experts [23]. However, certain human experts were more accurate than the computer-based models with one individual predicting 46 of 48 matches correctly. Perhaps the greater average accuracy of these computer-generated models can be attributed to the increased agreeability of the predictions across the different systems as this is associated with more accurate predictions [24,25,26]. The ability of computerised models to consistently and efficiently analyse data are certainly strengths of these systems [24].

Human judgements are often prone to biases, which can result in overconfidence by experts [27]. Such overconfidence, coupled with the public’s tendency to overlook the numerous inaccurate forecasts and celebrate the rare accurate ones, form the grounds of the bizarreness effect [3,4,5,6] and inflate claims of the accuracy of expert forecasts in sport [28]. This explains why bold predictions capture the public’s attention and disrupt memory of other events [6] as exemplified by SI’s Houston Astros prediction and the resulting impact on the public’s perception of SI’s predictive accuracy [1,2].

With greater access to performance statistics and other information than ever before, a relevant question is whether this surplus of data helps or hinders human forecasts [10,29,30,31]. Coincident with this increased access to data, there has been a greater focus on the value of ‘simple heuristics’ for improving the accuracy of decision-making and forecasting [32]. For instance, several studies have explored the availability heuristic—a phenomenon that postulates a well-recognised competitor (i.e., one which is most readily ‘available’ in our brains) is more likely to win compared to a less recognised one [10,31,32]. This work suggests having less information may be more beneficial when making decisions under uncertainty, although results in this area have been mixed [11,29,30,33]. The sociocultural implications and the way sport is constructed in society can thus influence individuals when making forecasts.

Despite the evidence presented above that may question the validity of experts, individuals continue to rely on expert opinions [30] presumably, at least in part, due to their access to useful (and often proprietary) information. However, it is important to critically examine ‘experts’ forecasting records to evaluate if the public should continue to deem these predictions as valuable. Furthermore, given that their ideas are released on behalf of large, seemingly reputable organisations, sports media outlets such as SI have the propensity to shape overall public opinion about the value and accuracy of sports forecasting. Given these implications, the predictive accuracy of popular media outlets is an area ripe for further exploration. SI is among the most distinguished magazines worldwide. Their inaugural issue in 1954 featured “Scouting Reports” and seasonal performance forecasts have continued to grow and evolve ever since (i.e., frequency, variables included, and time to prediction). A focused examination of SI’s predictive accuracy provides an opportunity for a unique case study of predictive accuracy in professional sports over a longer time frame than possible in events such as the World Cup of Soccer [34], World Cup of Rugby [10,23], and Wimbledon Tennis Championship [31]. One of the few studies to examine the predictive accuracy of season-long MLB forecasts found that SI and the New York Times made more accurate predictions than random guesses, except for the National League Conference winners [35]. Although informative, this study deserves re-assessment as the MLB has been expanded and re-aligned since this 1994 study. Therefore, the objective of this study was to assess the accuracy of SI’s pre-season forecasts of the divisional standings, conference standings, and championship winners of the four main North American sport leagues (the NFL, NBA, MLB, and NHL) over the past 30 years.

## 2. Materials and Methods

### 2.1. Procedures

Quantitative content analysis of the popularised magazine, SI was utilised as the primary research method. Content analysis refers to the objective, systematic, and quantitative analysis of communications content [36]. Pre-season predictions made by SI between 1988 and 2018 were collected from the magazine’s online repository. The repository consists of digitised versions of paper magazines inclusive of SI’s inaugural 1954 issue to the latest SI magazines from 2021. This archive can be freely accessed at https://vault.si.com/vault/archives (accessed on 14 August 2021). All four major North American sporting leagues- the NFL, NBA, MLB, and the NHL—were examined and predictions included the championship winner, runner-up, and score (for the NFL only), as well as divisional standings and conference winners where available. In years where SI did not explicitly state their prediction, inferences were made for each division based on their rankings of the conference (i.e., the team in each division with the highest predicted conference ranking was inferred to be the predicted divisional winner). This method was utilised for 8.1% of the data. Predictions were then compared to official results for the respective leagues. Throughout the process, the number of teams within each league, conference, and division was noted. Some data (13.7%) were missing from the dataset for reasons such as league lockouts, missing pages in the magazines, or unclear predictions.

### 2.2. Data Analysis

Several, largely descriptive, approaches were taken in the analyses. Data were analysed in Statistical Package for the Social Sciences (SPSS). Two areas of interest were highlighted—determining the absolute accuracy of SI’s predictions and the relative accuracy of their predictions (i.e., exactly how far the experts were off if they were incorrect). Initially, a simple binary score was calculated to determine the level of overall agreement for the predictions (i.e., was the prediction correct? Yes/No). The total number of correct forecasts within each division, conference, and league were collected, and following this, the average percentage of correct predictions was calculated for each domain to yield a comparable value (i.e., this number considered the total predictions made and controlled for elements such as the changing structural compositions of each league, lockouts, missing data and unclear predictions). Subsequently, comparisons between predicted vs. actual results for winner and runners-up provided information on relative accuracy. Predicted vs. actual results were calculated via discrepancy scores where the team’s predicted standing was subtracted from their actual standing. Thus, smaller discrepancy scores were associated with greater relative accuracy (i.e., the discrepancy between where the team actually finished is close to where SI predicted they would finish). Since the playoff rounds yielded ambiguous final standings (i.e., at the conclusion of each round, numerous teams are eliminated at once) a relative scale was created to clarify the actual results for the winner and runner-up predictions. The scale was as follows (1 = Won Championship, 2 = Lost in Championship, 3 = Lost in Third Round, 4 = Lost in Second Round, 5 = Lost in First Round, 6 = Did not Qualify for Playoffs). The average discrepancy scores for each division, conference, and league were then calculated, permitting comparisons between these categories.

As expected, the data violated the assumption of normality for the use of parametric statistics. Therefore, non-parametric Mann–Whitney *U* and Kruskal–Wallis H tests were used to compare groups. The exact significance level is reported where applicable, and the Monte Carlo permutation was used as a substitute if computational limits did not allow the exact significance to be calculated. For Kruskal–Wallis H Tests that met the significance level of 0.05, Mann–Whitney U Tests were utilised as a ‘post hoc’ test to further investigate the finding.

Due to the exploratory nature of this study, we used two-tailed tests for all comparisons. Furthermore, Bonferroni’s correction was applied to our alpha level to reduce the likelihood of Type 1 errors. However, given the small sample sizes, we focused on measures of effect size over indicators of statistical significance. Effect sizes for the Mann–Whitney U tests are in the form of Pearson’s correlation coefficient (*r*) and for Kruskal–Wallis tests as epsilon-squared (*E*^2^*_R_*) [37]. For the purposes of this study, small effect sizes were categorised as 0.2 or less, moderate effect sizes were categorised as 0.2–0.5 and large effect sizes were deemed anything 0.5 or greater.

The variables (number of correct predictions, average percentage of correct predictions, and average discrepancy score) were also totalled for the divisions and conferences within the league to provide a comparison between leagues (i.e., to determine whether SI made the most correct forecasts in the NFL, NBA, MLB, or NHL). For the winner and runner-up predictions for each league, we used Kruskal–Wallis H tests to compare across three time brackets (with approximately the same number of years in each bracket) to determine changes in the percentage of correct predictions (hereinafter referred to as “percent correct”) and discrepancy score. For both NFL predictions, both NBA predictions and the NHL Champion prediction, the time brackets were as follows: 1988–1997 vs. 1998–2007 vs. 2008–2018. SI made fewer predictions for the MLB champion, runner-up, and for the NHL runner-up and, as a result, their time brackets were as follows: 1996–2003 vs. 2004–2011 vs. 2012–2018. For the discrepancy scores, a Jonckheere–Terpstra Test of Trend was chosen to examine changes over time across these time brackets.

Due to changes in the sports leagues over the past 30 years, we attempted to standardise the accuracy values relative to the number of teams in the division/conference/league. The average number of teams per division/conference/league was calculated over the 30-year time frame, and these values were taken into account when examining the number of correct predictions and the average percentage of correct predictions in each respective category.

## 3. Results

Results from the Kruskal–Wallis and Mann–Whitney U tests are presented below and in Table 1. Descriptive statistics for each league are presented below (as well as in Table 2), followed by analyses comparing leagues (Table 3 and Table 4) and trends over time (Table 5, Table 6 and Table 7).

### 3.1. NFL

NFL league-wide data (i.e., winner vs. runner-up) revealed medium-sized effects but only the discrepancy scores displayed significant differences (see Table 1). Comparing the American Football Conference (AFC) to the National Football Conference (NFC) yielded medium effect sizes, with significantly higher discrepancy scores for the NFC compared to the AFC. Interestingly, the percent correct and discrepancy scores for the NFC did not significantly differ from the league winner category, despite the NFC having half as many teams as the entire NFL. Divisionally, non-significant small effect sizes were found when examining the AFC and NFC divisions, as well as when comparing the AFC to the NFC. Furthermore, as the average number of teams increased, percent correct decreased and discrepancy scores increased (i.e., SI was less accurate with larger groups). Divisional predictions showed significantly higher percent correct and lower discrepancy scores than conference and league-wide data. Moderate and small-to-moderate effect sizes were found when examining divisional vs. league-wide and conference-wide predictions, respectively.

Examining league winner forecasts over the last 30 years revealed small effect sizes for both percent correct and discrepancy scores, although none of these effects met the criteria for statistical significance (see Table 5). Additionally, the Jonckheere–Terpstra Test of Trend revealed small effect sizes and no discernible trends (see Table 7).

### 3.2. NBA

The NBA data (see Table 1) revealed non-significant small effects between the league winner and runner-up predictions. Small effect sizes were also seen when comparing the predictions for the Eastern Conference and Western Conference Champions. Non-significant small effects were also found when examining the NBA divisions, indicating that the division being predicted had little relation to SI’s resulting accuracy. SI was, however, significantly more likely to predict divisional winners compared to league and conference champions. Specifically, moderate, and small-to-moderate effect sizes were found when comparing SI’s accuracy in forecasting divisional champions to league and conference champions, respectively.

A small-to-moderate effect size was found when examining the discrepancy scores of NBA Champion predictions over the last 30 years. Significant differences and a large effect size were found between the 1988–1997 and 1998–2007 time brackets (see Table 5), where the latter had a larger median discrepancy score (see Table 6). A moderate effect size was found when comparing 1988–1997 vs. 2008–2018 and when comparing 1998–2007 and 2008–2018. Despite this, the Jonckheere–Terpstra Test indicated there were no significant trends (see Table 7).

### 3.3. MLB

Discrepancy scores of the MLB league winner and runner-up were significantly different with a large effect size (Table 1). At the conference level, non-significant small effects were noted between the American League (AL) and the National League (NL) winners. Divisionally, non-significant small effects were found, denoting that SI had no more success in one division than another. SI had significantly more success (i.e., higher percent correct and lower discrepancy scores) in predicting MLB divisional winners compared to league and conference champions, with moderate-to-large and small-to-moderate effects, respectively. Non-significant small effects were found for the forecasts of league winners compared to conference winners, indicating similar margins of error, despite conferences having half as many teams as the league.

Examining World Series Champion predictions via the Jonckheere–Terpstra test revealed a significant trend over time with a moderate-to-large effect size (see Table 7). Specifically, 1996–2003 had significantly smaller discrepancy scores than 2004–2011 with a large effect size between these two time periods. A non-significant small effect size was found between 2004–2011 and 2012–2018, suggesting an initial decrease in predictive accuracy (beginning in 2004) that has since remained consistent. Although no significant difference was found between the discrepancy scores of 1996–2003 and 2012–2018, there was a large effect size indicating SI’s predictive accuracy decreased over time.

### 3.4. NHL

Comparing the forecasted winner vs. runner-up in the NHL revealed no significant differences (see Table 1), and effect sizes were small-to-moderate for percent correct and small for discrepancy score. The Eastern conference had significantly larger discrepancy scores than the Western conference with a moderate-sized effect. Divisionally, non-significant small effects suggested the division being forecasted had little correlation with SI’s predictive accuracy. Furthermore, as the average number of teams decreased, predictive accuracy increased. For example, percent correct was significantly higher and discrepancy scores significantly lower for divisions compared to league-wide predictions with moderate effect sizes. Comparisons between divisional and conference forecasts indicated a significant moderate effect size for discrepancy score but non-significant small effects for percent correct.

Examining SI’s predictions for the Stanley Cup winner over time (see Table 5) yielded small effects for percent correct and discrepancy scores, implying little correlation between time and SI’s predictive accuracy. Overall, no significant differences were found between time brackets, and the Jonckheere–Terpstra test confirmed there were no distinct trends over time (see Table 7).

### 3.5. Comparing across Leagues

An analysis combining both league-wide predictions (i.e., NFL championship winner and runner-up vs. NBA championship winner and runner-up, etc.), revealed the NBA had the largest percent correct (see Table 3). Moderate effect sizes and significant differences were revealed between the NBA and NFL, as well as between the NBA and MLB. A non-significant small effect was found when comparing the NBA to NHL. SI also experienced the greatest relative accuracy in the NBA with significantly lower discrepancy scores than in the other three leagues and moderate effect sizes. Non-significant small effects were found when comparing the other leagues on both percent correct and discrepancy scores (see Table 3).

The percent correct and discrepancy scores for the divisions and conferences within each league were combined to yield statistics that could be used for comparison across leagues. Generally, at the conference level, small effects and no significant differences were found for percent correct (see Table 3). SI experienced the greatest relative accuracy with the NBA at the conference level. However, the only significant difference was found between the NBA and MLB and this comparison yielded a moderate effect size.

Divisionally, there were significant differences in percent correct and discrepancy scores (see Table 3) although effect sizes were small. Again, SI’s divisional NBA predictions had significantly higher percent correct than divisional predictions for the NFL and NHL. SI also experienced the greatest relative accuracy with their divisional NBA predictions, which had significantly lower discrepancy scores than other leagues. An analysis of all predictions found SI’s overall percent correct was 32.8%.

## 4. Discussion

The use of technology and the internet has allowed for greater forecast examination, but only in recent years. Analyses of SI’s predictive accuracy over the last 30 years revealed several intriguing findings. These results help to critically examine the forecasting accuracy of ‘experts’ and how their predictions shape sociocultural notions of sport in society.

First, there was a clear, and unsurprising, trend showing increased forecasting accuracy when the average number of teams was the lowest (i.e., divisions had significantly higher percent correct and lower discrepancy scores than conference and league-wide predictions). However, non-significant small effect sizes were found between divisions, indicating that the division being forecasted had little relation to SI’s resulting predictive accuracy. This was somewhat surprising as certain divisions (e.g., the AFC East in football, the AL East in baseball) are known for ‘powerhouse’ organisations who have historically been consistent contenders for divisional championships. Perhaps expanding markets of teams in these divisions such as the Tampa Bay Rays (who finished in last place every year from 1998–2007, yet since 2008 have had a winning record in eight years) have led to more uncertainty in these perceived ‘easy-to-predict’ divisions. Alternatively, if there is no clear divisional favourite, the small pool of teams to choose from may increase the likelihood of correct predictions. Finally, each league has implemented numerous structural changes over the last 30 years (i.e., divisional adjustments, expansion teams). This may also impact why SI had no more success in predicting the winners of one division compared to another, as continual divisional re-alignments may hinder any emerging trends.

### 4.1. SI Accuracy Is Greatest in the NBA

At the divisional, conference, and league levels, SI had the greatest predictive success in the NBA. This may reflect how basketball is structured in relation to other sports. For example, with more scoring attempts per game (i.e., due to a ‘shot clock’ that requires teams to shoot the ball every 24 s), the likelihood of random outcomes decreases as the variance of points regress towards a mean value determined by the ‘talent’ on a given team [38]. Moreover, the NBA playoffs are structured as four, best-of-seven series. As a result, lengthening the playoff series reduces the likelihood of a weaker team ‘upsetting’ the stronger team [39]. In comparison, playoff formats of other leagues rely on single elimination games (e.g., the NFL) or shorter series (e.g., the MLB). Similarly, NBA rosters possess the fewest athletes, which further reduces uncertainty by limiting the potential for variance (i.e., fewer players to project).

It is also likely that greater prediction accuracy in the NBA is associated with the league’s financial structure, which differs from other North American leagues in that it operates under a ‘soft’ salary cap (i.e., franchise payrolls are limited yet some exceptions are permitted). The salary cap was implemented in 1984 with the hopes of increasing competitive balance. However, it has been largely ineffective as the lack of parity in the NBA has remained consistent over the last 30 years [38,40] seemingly making the outcomes easier to predict than other leagues. It is argued the large exemptions to the cap have been detrimental to league parity [41]. For example, the NBA is renowned for dynasties such as the Boston Celtics, Chicago Bulls, and more recently the Golden State Warriors. While other leagues also possess dominating organisations, the NBA differs as its “Larry Bird Exemption” is a clause under the ‘soft’ salary cap that allows teams to re-sign one of their own players without regard to the salary cap [41,42]. These ‘loopholes’ in the salary cap damage the competitive balance as large-market franchises can match outside offers and keep their star players together for successive seasons [41]. In comparison, the NHL’s revised Collective Bargaining Agreement (CBA) implemented in 2005 has decreased an organisation’s capacity to “hold-together” championship teams whereby no NHL team has won the Stanley Cup more than two years in a row [42]. Even prior to the new CBA, a team winning the Stanley Cup in more than two consecutive years has only happened twice in the post-expansion era (1967 to present). Similarly, the NFL illustrates greater competitive balance as a team has yet to win three consecutive Super Bowl Titles [43]. While the primary mechanisms explaining these effects are unknown, the existence of these trends suggests a range of additional factors that may explain SI’s greater predictive accuracy in the NBA.

### 4.2. No Changes in Prediction Accuracy over Time

In general, results indicated no significant differences or meaningful effects in SI’s predictive accuracy over time. This was somewhat surprising considering the increased attention given to the use of predictive analytics in short- and long-term sport forecasting. However, despite advancements in analytics over the last 30 years, emphasis has also been placed on increasing competitive balance within leagues in order to attract fans, prevent bankruptcy and discourage the creation of rival leagues [43,44,45,46]. Evidence suggests that parity has generally improved for most leagues since the 1990–1999 decade [38]; and as a consequence, accurate forecasting may have become more difficult (i.e., a more ‘balanced’ league with numerous contenders is theoretically more difficult to predict).

In an effort to achieve competitive balance, leagues have introduced various redistribution mechanisms (e.g., revenue-sharing, reverse-order drafts, free-agency, and salary caps). Ultimately, these measures must be addressed as their proposed benefits to competitive balance [38,41,42,46] may influence forecasting ability. However, their effectiveness is largely unknown as recent increases in competitive balance may not be a direct result of these redistribution mechanisms (i.e., causation cannot be inferred). For example, North American sports also use unbalanced schedules (i.e., teams do not play opponents the same number of times), a format that traditionally produces more uncertainty [47], which also may have contributed to greater league parity. Traditional redistribution mechanisms include revenue-sharing and numerous studies have explored the effects of revenue-sharing with mixed results [48,49,50,51,52,53,54]. Additionally, the effectiveness of the ‘reverse-order’ draft is also largely unknown as it is argued that adding only one player may not be impactful [55]. Free agency is another redistribution mechanism implemented by all four leagues, yet it is uncertain whether it helps or hinders predictive accuracy. This clause allows for player mobility yet may also permit the formation of ‘super-teams’. Finally, salary caps (which are designed to limit large-market teams from signing all the elite athletes) [56] are another redistribution mechanism that may impact predictive accuracy. While their effects are also ambiguous [38,41,46] the NHL and NFL are argued to have greater parity due to their ‘hard’ salary caps [38] compared to the NBA’s ‘soft’ salary cap and the MLB’s luxury tax (i.e., a surcharge for teams that exceed a pre-determined payroll amount) [41]. Overall, it is unclear how these redistribution mechanisms may have affected the predictive accuracy in our analyses. The possible direct and interactive effects provide a host of intriguing areas of further work.

While SI’s forecasting accuracy remained largely consistent over time, there was a trend of decreasing accuracy in the MLB. Specifically, 1996–2003 had significantly lower discrepancy scores than in 2004–2011. Although this suggests that SI’s accuracy in predicting World Series Champions has decreased over time, the dominance of the New York Yankees, who won four league titles during 1996–2003, needs to be considered. Nonetheless, as previously noted, parity in MLB has generally grown over time [48,57] creating greater difficulty in forecasting the World Series Champions. Other factors may also be especially relevant for MLB forecasts (e.g., performance-enhancing drugs; MLB’s 182-game regular season) but are largely unaccounted for in SI’s pre-season predictions. Last, although SI’s remarkable three-year out Houston Astro’s prediction sparked this study, recent evidence revealed unfair play on the Astro’s behalf [58] and this must be acknowledged as a potential confounding factor in SI’s and our analyses.

### 4.3. Limitations and Future Research

This study is one of few to examine long-term forecasting accuracy, using data from a popular American media outlet. Although interesting results were discovered, the study had some notable limitations. First, 30 years of data, although impressive as a longitudinal data source, provided a relatively small number of data points, some of which were missing due to reasons such as league lockouts, missing pages in the magazine, or if SI had not made a prediction that year. As a result of this small sample size, the statistical tests conducted were generally crude and conservative tests, increasing the possibility of type II error. While the purpose of this research project was to present an initial exploration into the SI’s forecasting accuracy, future studies may seek to use more rigorous statistical tests, as data becomes more readily available (i.e., as the forecasting pool grows when SI makes pre-season predictions in subsequent years). In an effort to increase sample size, future studies may also seek to compare the forecasting accuracy of multiple news outlets (i.e., to determine if certain sport media outlets are more successful than others) or compare the predictive accuracy of media outlets to betting odds or other forecasting forums. Finally, SI’s “team of editors” who made the prediction each year was inconsistent (i.e., the team changed over time), which confounded our ability to discern predictive accuracy over time. Future work could overcome this limitation by examining a single forecaster, for example, a notable sports commentator, over time.

Additionally, the present study could be enhanced by comparing SI’s pre-season predictions to other ‘futures betting markets’ or sporting prediction magazines. Positioning SI’s projections against different measures of across-season competitive balance estimates could help to provide a benchmark for SI’s abilities to forecast and highlight the strengths and weaknesses of each source’s respective forecasting abilities. Using the futures betting market could be an especially fruitful comparison as returns could be calculated to see if following SI’s predictions make money in this time frame.

It is also worth considering that forecasting accuracy is a small segment in the entirety of sports culture and mass media communications. The changing dynamics within the sociocultural vehicle of sports and how SI represents those values must be considered when examining their predictive accuracy. Several other studies have analysed periodicals over time and noted how their content is impacted by the time period in which they are published [59,60].

Last, the authors were unable to determine how SI predictions were/are made. For this reason, future work could benefit from understanding whether SI forecasts were the result of one person’s gut (i.e., tipsters/pundits), an aggregation of several people, whether there was some sort of statistical model being used by SI and/or some variation of all the above.

## 5. Conclusions

This study presented an initial exploration into SI’s pre-season forecasting accuracy for the four major North American sport championships over the last 30 years. While results varied across leagues, SI was generally more successful at predicting divisional winners compared to conference and league champions. Interestingly, in all leagues, it was found that SI was not significantly better at predicting certain divisions compared to others. A direct comparison of leagues revealed SI experienced the greatest predictive accuracy in the NBA. Furthermore, an examination of 30 years of predictions revealed no distinct trends over time, yet there is some evidence to suggest that forecasting accuracy for the MLB may have decreased. This study suggests that despite technological advancements, expert opinions still carry some value in modern-day sport forecasting. Findings from this study highlight that expert opinions should be viewed in relation to the sociocultural lens of sport, and help to shape the way society views sport as a whole. Although greater research is required to fully optimise predictive success in North American sporting leagues, this study presents a valuable baseline analysis of SI’s forecasting accuracy.

## Figures and Tables

**Table 1:** Results from Kruskal–Wallis and Mann–Whitney U Tests for each league.

	Percent Correct			Discrepancy Score		
Comparison	U/H	*p*	R	U/H	*p*	R
NFL						
W vs. RU	434.00	0.24	−0.22	344.50	0.05	−0.25
AFC vs. NFC	157.50	0.07	−0.33	101.00	0.002	−0.47
W vs. RU vs. AFC vs. NFC	17.24	0.001	0.17	15.64	0.001	0.15
W vs. AFC	233.00	0.02	−0.34	161.50	0.001	−0.43
W vs. NFC	325.00	1.00	−0.002	274.00	0.34	−0.13
RU vs. AFC	201.50	<001	−0.51	200.50	0.02	−0.33
RU vs. NFC	294.50	0.16	−0.24	225.50	0.06	−0.26
L vs. C vs. D	35.34	<001	0.11	64.10	<001	0.20
L vs. C	1055.00	0.006	−0.28	1164.50	0.36	−0.09
L vs. D	41,430.00	<001	−0.34	2679.00	<001	−0.45
C vs. D	3683.00	0.02	−0.15	2775.00	<001	−0.27
AFC E vs. W vs. N vs. S vs. C	1.95	0.75	0.02	1.24	0.88	0.01
NFC E vs. W vs. N vs. S vs. C	5.56	0.23	0.05	6.80	0.15	0.06
AFC divs vs. NFC divs	5335.00	0.10	−0.12	5367.00	0.13	−0.10
NBA						
W vs. RU	420.00	0.78	−0.01	400.00	0.45	−0.01
East vs. West	264.00	0.77	−0.08	285.00	0.95	−0.01
W vs. RU vs. East vs. West	0.85	0.84	0.01	0.83	0.85	0.01
L vs. C vs. D	16.61	<001	0.07	23.48	<001	0.09
L vs. C	1380.00	0.69	−0.04	1328.00	0.48	−0.07
L vs. D	3170.00	<001	−0.25	2790.00	<001	−0.31
C vs. D	2682.00	0.007	−0.20	2522.50	0.001	−0.23
Atlantic vs. Central vs. SE	0.74	0.65	0.01	0.88	0.64	0.01
SW vs. NW vs. Pacific vs. MW	2.20	0.53	0.03	1.58	0.66	0.02
East divs vs. West divs	2336.00	0.17	−0.13	2330.00	0.13	−0.12
MLB						
W vs. RU	231.00	1.00	−0.09	102.00	0.001	−0.50
AL vs. NL	182.50	1.00	−0.05	169.50	0.57	−0.09
W vs. RU vs. AL vs. NL	4.67	0.20	0.06	7.78	0.05	0.09
W vs. AL	175.00	0.09	−0.29	173.50	0.24	−0.18
W vs. NL	174.50	0.16	−0.25	201.00	0.84	−0.03
RU vs. AL	185.00	0.23	−0.21	181.00	0.32	−0.15
RU vs. NL	184.00	0.39	−0.17	151.00	0.13	−0.24
L vs. C vs. D	28.44	<001	0.11	52.07	<001	0.21
L vs. C	718.50	0.06	−0.23	799.50	0.59	−0.06
L vs. D	2163.00	<001	−0.34	1467.50	<001	−0.44
C vs. D	2443.50	0.007	−0.19	1686.50	<001	−0.34
AL East vs. Central vs. West	2.48	0.28	0.03	2.88	0.24	0.04
NL East vs. Central vs. West	1.80	0.44	0.02	2.87	0.24	0.04
AL divs vs. NL divs	3114.00	0.28	−0.10	3179.50	0.36	−0.07
NHL						
W vs. RU	278.50	0.06	−0.27	313.00	0.53	−0.09
East vs. West	180.00	0.60	−0.11	128.50	0.05	−0.31
W vs. RU vs. East vs. West	5.37	0.14	0.06	5.75	0.13	0.06
L vs. C vs. D	11.77	0.002	0.06	36.43	<001	0.17
L vs. C	955.00	0.29	−0.12	1027.00	0.78	−0.03
L vs. D	2292.50	0.001	−0.25	1440.50	<001	−0.44
C vs. D	1960.00	0.09	−0.14	1421.00	<001	−0.30
Atlantic vs. Met vs. NE vs. SE	1.34	0.71	0.02	3.87	0.28	0.07
Central vs. NW vs. Pacific	3.42	0.19	0.06	2.62	0.27	0.05
East divs vs. West divs	1363.00	0.06	−0.19	1403.50	0.10	−0.15

Note: W = League Winner; RU = League Runner-Up; AFC = American Football Conference; NFC = National Football Conference; L = League-Wide forecasts; C = Conference-Wide forecasts; D = Divisional forecasts; E = East; W = West; N = North; S = South; C = Central; SE = South East; SW = South West; NW = North West; MW = Mid-West; AL = American League; NL = National League; Met = Metropolitan; NE = North East.

**Table 2:** Descriptive Statistics for each league.

Category	Avg. N	Avg. % Correct	Mdn. Discrepancy Score
NFL			
Winner	30.8	0.1	−4.0
Runner-Up	30.8	0	−2.0
AFC	15.4	0.4	−1.0
NFC	15.3	0.1	−3.0
AFC East	4.5	0.6	0
AFC North	4.0	0.4	−1.0
AFC West	4.5	0.5	−1.0
AFC South	4.0	0.5	−1.0
AFC Central	4.7	0.6	0
NFC East	4.5	0.3	−1.0
NFC North	4.0	0.5	0
NFC West	4.2	0.5	0
NFC South	4.0	0.3	−1.0
NFC Central	5.0	0.2	−1.0
League	30.8	0.05	−2.0
Conference	15.4	0.2	−2.0
Division	4.3	0.4	−1.0
NBA			
Winner	29.0	0.4	−1.5
Runner-Up	29.0	0.3	−1.5
Eastern Conference	14.6	0.3	−1.0
Western Conference	14.4	0.4	−1.0
Atlantic	5.9	0.7	0
Central	6.3	0.7	0
South East	5.0	0.6	0
South West	5.0	0.7	0
North West	5.0	0.5	−1.0
Pacific	6.0	0.6	0
Mid-West	6.8	0.4	−1.0
League	29.0	0.3	−1.5
Conference	14.5	0.4	−1.0
Division	5.7	0.6	0
MLB			
Winner	29.0	0.04	−4.0
Runner-Up	29.0	0.1	−2.0
AL	14.2	0.2	−3.0
NL	14.9	0.2	−4.0
AL East	5.4	0.5	0
AL Central	5.0	0.6	0
AL West	4.8	0.4	−1.0
NL East	5.2	0.5	−0.5
NL Central	5.6	0.5	−1.0
NL West	5.1	0.3	−1.0
League	29.0	0.1	−3.0
Conference	14.5	0.2	−3.0
Division	5.2	0.5	−1.0
NHL			
Winner	27.9	0.2	−3.0
Runner-Up	27.9	0.04	−2.5
Eastern Conference	15.0	0.2	−3.5
Western Conference	14.3	0.3	−1.5
Atlantic	6.1	0.3	−1.0
Metropolitan	8.0	0.2	−2.0
North East	5.3	0.3	−1.0
South East	4.9	0.4	−1.0
Central	5.6	0.4	−1.0
North West	4.9	0.7	0
Pacific	5.8	0.5	−1.0
League	27.9	0.1	−3.0
Conference	14.6	0.2	−2.0
Division	5.8	0.4	−1.0

Note: Mdn = Median; League = all League-Wide predictions (e.g., forecasts for league winner and league runner up); Conference = predictions from both conferences (e.g., the AFC and NFC); Division = predictions from all the divisions within the league.

**Table 3:** Comparison across leagues at the League, Conference, and Divisional levels.

Comparison	Percent Correct			Discrepancy Score		
	U/H	*p*	R	U/H	*p*	R
L_NFL_ vs. L_NBA_ vs. L_MLB_ vs. L_NHL_	22.36	<001	0.10	22.21	<001	0.10
NFL vs. NBA	1330.00	<001	−0.36	1091.50	<001	−0.36
NFL vs. MLB	1337.00	0.69	−0.04	1319.50	0.77	−0.03
NFL vs. NHL	3427.50	0.11	−0.17	3431.00	0.35	−0.09
NBA vs. MLB	970.00	0.002	−0.31	753.50	<001	−0.37
NBA vs. NHL	1300.00	0.03	−0.21	1035.00	0.001	−0.31
MLB vs. NHL	1069.50	0.33	−0.13	1019.00	0.28	−0.11
C_NFL vs_. C_NBA_ vs. C_MLB_ vs. C_NHL_	3.15	0.37	0.02	11.84	0.01	0.07
NFL vs. NBA	870.00	0.18	−0.15	723.50	0.02	−0.25
NFL vs. MLB	813.00	1.00	−0.01	717.50	0.33	−0.11
NFL vs. NHL	830.00	1.00	−0.01	836.00	0.97	−0.004
NBA vs. MLB	801.00	0.17	−0.15	576.00	0.002	−0.34
NBA vs. NHL	840.00	0.25	−0.13	690.00	0.02	−0.25
MLB vs. NHL	765.00	1.00	−0.02	661.50	0.24	−0.13
D_NFL_ vs. D_NBA_ vs. D_MLB_ vs. D_NHL_	13.75	0.004	0.02	12.57	0.005	0.02
NFL vs. NBA	13,351.00	0.002	−0.16	13,200.00	0.002	−0.16
NFL vs. MLB	17,621.00	0.54	−0.03	18,035.00	0.82	−0.01
NFL vs. NHL	12,304.00	0.56	−0.03	12,516.00	0.76	−0.02
NBA vs. MLB	10,498.00	0.02	−0.13	10,194.00	0.01	−0.15
NBA vs. NHL	6737.00	0.001	−0.20	6749.00	0.002	−0.19
MLB vs. NHL	8947.00	0.27	−0.07	9324.00	0.63	−0.03

Note: L = League-Wide predictions (e.g., the forecasts for League-Winner and Runner-Up); C = Conference-Wide predictions (e.g., the forecasts for both conferences); D = Divisional predictions for each league.

**Table 4:** Summary of statistics across leagues.

Category	League	Avg. N	Avg. % Correct	Mdn. Discrepancy Score
League−Wide	NFL	30.7	0.05	−2.0
League−Wide	NBA	29.0	0.3	−1.5
League−Wide	MLB	29.0	0.1	−3.0
League−Wide	NHL	27.9	0.1	−3.0
Conference−Wide	NFL	15.4	0.2	−2.0
Conference−Wide	NBA	14.5	0.4	−1.0
Conference−Wide	MLB	14.5	0.2	−3.0
Conference−Wide	NHL	14.6	0.2	−2.0
Divisional	NFL	4.3	0.4	−1.0
Divisional	NBA	5.7	0.6	0
Divisional	MLB	5.2	0.5	−1.0
Divisional	NHL	5.8	0.4	−1.0

**Table 5:** Results from Kruskal–Wallis and Mann–Whitney U Tests for each league over time.

Comparison		Percent Correct			Discrepancy Score		
	League	U/H	*p*	R	U/H	*p*	R
W vs. W vs. W	NFL	2.32	0.29	0.08	0.36	0.84	0.01
RU vs. RU vs. RU	NFL	0	1.00	0	0.99	0.62	0.03
W vs. W vs. W	NBA	5.27	0.09	0.18	6.82	0.03	0.23
1998–2007 vs. 2008–2018	NBA	-	-	-	29.50	0.12	−0.35
1988–1997 vs. 1998–2007	NBA	-	-	-	17.00	0.01	−0.58
1988–1997 vs. 2008–2018	NBA	-	-	-	38.50	0.40	−0.21
RU vs. RU vs. RU	NBA	2.76	0.40	0.09	2.32	0.34	0.08
W vs. W vs. W	MLB	1.75	0.42	0.08	7.02	0.02	0.33
2004–2011 vs. 2012–2018	MLB	-	-	-	23.50	1.00	−0.04
1996–2003 vs. 2004–2011	MLB	-	-	-	8.50	0.02	−0.62
1996–2003 vs. 2012–2018	MLB	-	-	-	12.00	0.05	−0.50
RU vs. RU vs. RU	MLB	3.67	0.30	0.18	5.69	0.05	0.27
W vs. W vs. W	NHL	4.78	0.10	0.17	3.54	0.17	0.13
RU vs. RU vs. RU	NHL	2.14	0.64	0.10	1.93	0.40	0.09

Note: W = League Winner; RU = League Runner-Up.

**Table 6:** Descriptive statistics over time.

Time Bracket	League	Avg. N	Avg. Percent Correct	Mdn. Discrepancy Score
1988–1997	NFL	28.6	0.2	−3.0
1998–2007	NFL	31.5	0.1	−3.5
2008–2018	NFL	32.0	0	−4.0
1988–1997	NBA	27.6	0.6	0
1998–2007	NBA	29.4	0.1	−3.0
2008–2018	NBA	30.0	0.4	−1.0
1996–2003	MLB	29.5	0.1	−3.0
2004–2011	MLB	30.0	0	−5.0
2012–2018	MLB	30.0	0	−5.0
1988–1997	NHL	23.9	0	−4.0
1998–2007	NHL	29.0	0.4	−1.0
2008–2018	NHL	30.2	0.3	−2.0

**Table 7:** Jonckheere–Terpstra Test of Trend Results.

Comparison	Discrepancy Score			
	League	J	*p*	R
W vs. W vs. W	NFL	175.00	0.58	0.10
RU vs. RU vs. RU	NFL	160.50	0.99	0.003
W vs. W vs. W	NBA	174.00	0.35	0.17
RU vs. RU vs. RU	NBA	144.50	0.82	−0.04
W vs. W vs. W	MLB	115.00	0.02	0.48
2004–2011 vs. 2012–2018	MLB	23.50	1.00	−0.04
1996–2003 vs. 2004–2011	MLB	47.50	0.02	0.62
1996–2003 vs. 2012–2018	MLB	44.00	0.08	0.50
RU vs. RU vs. RU	MLB	106.00	0.12	0.34
W vs. W vs. W	NHL	111.50	0.25	−0.22
RU vs. RU vs. RU	NHL	77.00	0.84	−0.05

Note: W = League Winner; RU = League Runner-Up.

## Data Availability

Data can be made available upon request. All requests for data can be made directly to krobinso@yorku.ca.
